# Patterns of abundance, chromosomal localization, and domain organization among c-di-GMP-metabolizing genes revealed by comparative genomics of five alphaproteobacterial orders

**DOI:** 10.1186/s12864-022-09072-9

**Published:** 2022-12-16

**Authors:** Sonja Koppenhöfer, Andrew S. Lang

**Affiliations:** Department of Biology, Memorial University of Newfoundland, St. John’s, Newfoundland and Labrador Canada

**Keywords:** Phosphodiesterase, Diguanylate cyclase, DNA replication, Cell cycle, HD-GYP, GGDEF, EAL

## Abstract

**Background:**

Bis-(3′-5′)-cyclic dimeric guanosine monophosphate (c-di-GMP) is a bacterial second messenger that affects diverse processes in different bacteria, including the cell cycle, motility, and biofilm formation. Its cellular levels are controlled by the opposing activities of two types of enzymes, with synthesis by diguanylate cyclases containing a GGDEF domain and degradation by phosphodiesterases containing either an HD-GYP or an EAL domain. These enzymes are ubiquitous in bacteria with up to 50 encoded in some genomes, the specific functions of which are mostly unknown.

**Results:**

We used comparative analyses to identify genomic patterns among genes encoding proteins with GGDEF, EAL, and HD-GYP domains in five orders of the class Alphaproteobacteria. GGDEF-containing sequences and GGDEF-EAL hybrids were the most abundant and had the highest diversity of co-occurring auxiliary domains while EAL and HD-GYP containing sequences were less abundant and less diverse with respect to auxiliary domains. There were striking patterns in the chromosomal localizations of the genes found in two of the orders. The Rhodobacterales’ EAL-encoding genes and Rhizobiales’ GGDEF-EAL-encoding genes showed opposing patterns of distribution compared to the GGDEF-encoding genes. In the Rhodobacterales, the GGDEF-encoding genes showed a tri-modal distribution with peaks mid-way between the origin (*ori*) and terminus (*ter*) of replication and at *ter* while the EAL-encoding genes peaked near *ori*. The patterns were more complex in the Rhizobiales, but the GGDEF-encoding genes were biased for localization near *ter*.

**Conclusions:**

The observed patterns in the chromosomal localizations of these genes suggest a coupling of synthesis and hydrolysis of c-di-GMP with the cell cycle. Moreover, the higher proportions and diversities of auxiliary domains associated with GGDEF domains and GGDEF-EAL hybrids compared to EAL or HD-GYP domains could indicate that more stimuli affect synthesis compared to hydrolysis of c-di-GMP.

**Supplementary Information:**

The online version contains supplementary material available at 10.1186/s12864-022-09072-9.

## Introduction

Bis-(3′-5′)-cyclic dimeric guanosine monophosphate (c-di-GMP) is a second messenger that was first described for its role in regulating cellulose biosynthesis in *Gluconacetobacter xylinus* [1, 2], but which is now recognized as near-ubiquitous and affecting a large variety of processes in bacteria [3, 4]. Cellular concentrations of c-di-GMP are regulated in response to internal and external stimuli, and the resulting changes can be part of bacterial adaptation to changes in their environment [5]. The cellular levels of c-di-GMP are controlled by two groups of enzymes with opposing activities, where it is synthesized by diguanylate cyclases (DGCs) and degraded by c-di-GMP-specific phosphodiesterases (PDEs). DGCs have conserved GGDEF domains and synthesize c-di-GMP from two molecules of guanosine triphosphate (GTP) [6]. There are two distinct types of PDEs, with either EAL or HD-GYP domains, that degrade c-di-GMP. Both types are able to break c-di-GMP into the linear 5′-phosphoguanylyl (3′-5′) guanosine (pGpG) dinucleotide [7, 8] which is then further broken down to two molecules of guanosine monophosphate (GMP) by an oligo-ribonuclease [9, 10]. PDEs of the HD-GYP type can also break c-di-GMP into two GMPs in one step [11, 12]. In addition, there are hybrid proteins that have both GGDEF and EAL domains and thus represent a “biochemical conundrum”. It has been suggested that these proteins can switch between synthesis and hydrolysis of c-di-GMP [13], with a protein’s activity controlled by, for example, phosphorylation status [14] or dimerization [15]. However, it is also possible that one of the domains is not enzymatically functional. Based on the proteins characterized in detail, the most common scenarios are that only the EAL domain is functional or both domains are functional [16].

C-di-GMP levels can be controlled via transcriptional and translational regulation of gene expression, or through post-translational modification of the synthesis and degradation enzymes as a quicker response. Auxiliary domains can be present on the enzymes and include sensory, signalling and protein binding domains, and these can allow for rapid adaptation [17]. Cellular changes in c-di-GMP concentration can result from a variety of input and output signals that are detected by the enzymes or their regulators and that affect the production or degradation of c-di-GMP [18]. An analysis of genomic sequences from different bacterial phyla found that members of the phylum Proteobacteria encode the highest numbers of c-di-GMP-modulating enzymes [18].

In the Alphaproteobacteria, c-di-GMP has been examined for its role in many different processes, such as the symbiosis of *Sinorhizobium meliloti* with plant roots [19] and related to its effects on the regulatory network associated with the transcriptional regulator CtrA [20], which is highly conserved in this class [21].The CtrA phosphorelay consists of the histidine kinase CckA, the phosphotransferase ChpT and the transcriptional regulator CtrA [22]. It has been suggested that its ancestral role in alphaproteobacteria was related to the control of motility and recombination [23, 24], but there has also been work establishing a link between this phosphorelay and c-di-GMP with respect to regulation of the cell cycle and cell differentiation in *Caulobacter crescentus* [20, 25] and gene transfer agent (GTA) production in *Rhodobacter capsulatus* and *Dinoroseobacter shibae* [26, 27]. C-di-GMP affects the CtrA phosphorelay directly through effects on the enzymatic activity of CckA, which changes the phosphorylation level of CtrA and thus its activity [28, 29]. The concentration of c-di-GMP also appears to be affected by CtrA because loss of CtrA results in changes in the transcript levels of genes encoding c-di-GMP-metabolizing enzymes [30].

The chromosomal positioning of genes can affect their functions in different ways and have effects on multiple cellular processes. For example, gene location can influence the spatial distribution of proteins within cells due to transcription-coupled translation [31]. Positioning can also have effects with respect to the cell cycle because genes that are close to the origin of replication (*ori*) are replicated earlier and are therefore temporarily present in higher copies than genes that are closer to the terminus of replication (*ter*) [32]. An example where this has important implications was found in *Bacillus subtilis*, where it was shown that the temporal copy number imbalances due to the opposite localization of genes encoding members of a regulatory network influenced its output [33, 34]. Additionally, gene location can influence expression due to the state of DNA methylation through the cell cycle. The partially replicated portions of the chromosome are hemi-methylated during replication starting at *ori*, and methylation status can affect regulatory protein binding and transcription [35]. For example, and directly related to regulatory systems already discussed above, one of the promoters where transcription of *ctrA* initiates is only activated in the hemi-methylated state in *C. crescentus* [36]. It seems likely there are additional and broader implications of gene location related to CtrA because a previous analysis also showed that numerous genes that are connected to CtrA have conserved chromosome positions in members of the Alphaproteobacteria [37].

C-di-GMP-modulating enzymes are broadly distributed in phylogenetically and metabolically diverse bacteria. They are also very diverse with respect to their roles and regulation, with a wide range of stimuli affecting c-di-GMP levels, and only a small proportion of the total diversity of these enzymes has been characterized in detail [38]. Therefore, we were interested in identifying any underlying genomic properties that these enzymes might share. We performed a comparative analysis of sequences containing GGDEF, EAL, and HD-GYP domains from five orders of the Alphaproteobacteria, the Rhodospirillales, Sphingomonadales, Rhodobacterales, Rhizobiales and Caulobacterales. We identified the auxiliary domains present with these c-di-GMP-metabolizing domains and attempted to identify patterns regarding enzyme occurrences, distributions, and chromosomal localizations.

## Methods

### Dataset

Protein sequences with identified EAL (PF00563), GGDEF (PF00990) or HD (PF01966) domains from genomes of bacteria within five orders of the Alphaproteobacteria (Rhodospirillales, Sphingomonadales, Rhodobacterales, Rhizobiales and Caulobacterales) were downloaded from the EMBL website on 6 August 2020 (GGDEF: http://pfam.xfam.org/family/PF00990#tabview=tab7; EAL: http://pfam.xfam.org/family/PF00563#tabview=tab7; HD: http://pfam.xfam.org/family/PF01966#tabview=tab7) [39]. Proteins with the HHExxxxxGYP motif from within the HD sequences were then selected and considered PDEs while the remaining HD sequences were considered auxiliary domains if they co-occurred with a c-di-GMP-metabolizing domain. Proteins with both EAL and GGDEF domains were placed in their own group (GGDEF_EAL).

All analyses were done in R version 4.0.3 with the appropriate packages as needed (Table S1).

### Organism, domain, and genomic annotations

Sequence identifiers were extracted from the EMBL fasta files and used to access the respective organism information from UniProt (e.g., https://www.uniprot.org/uniprot/V4RSF5.txt) or EBI (e.g., https://www.ebi.ac.uk/ena/browser/api/summary/PNQ76602) [40]. The identifiers were also used to withdraw the domain information from Pfam (e.g., http://pfam.xfam.org/protein/A0A0N0K049#tabview=tab0). Domain annotations could not be withdrawn for all sequences due to inconsistent html path formatting, which reduced the dataset (Table 1). The identifiers were also used to obtain the NCBI protein identifiers from UniProt (e.g., https://www.uniprot.org/uniprot/A0A0N0K3V8.txt). Due to inconsistencies some sequences have different version numbers (e.g., https://www.uniprot.org/uniprot/A0A0N0K3V8.txt?version=1) and only version 1 was subsequently considered in such cases. All sequence identifiers and html paths can be found in Table S2. The NCBI identifiers were used to obtain genomic information from the gff and fasta files, downloaded from NCBI in GenBank format.

**Table 1:** Genomes, genera, and species/strains available for analyses

Order	Closed genomes	One unambiguously identified *ori*	C-di-GMP-metabolizing domains	By genera^a^	By species or strains
Rhodospirillales	132	75	EAL	42	62
			GGDEF	47	71
			GGDEF_EAL	48	74
			HD-GYP	23	35
Sphingomonadales	27	17	EAL	22	145
			GGDEF	25	172
			GGDEF_EAL	26	174
			HD-GYP	8	18
Rhodobacterales	187	69	EAL	123	308
			GGDEF	132	333
			GGDEF_EAL	121	278
			HD-GYP	11	16
Rhizobiales	424	133	EAL	77	227
			GGDEF	90	257
			GGDEF_EAL	93	266
			HD-GYP	38	105
Caulobacterales	44	8	EAL	5	20
			GGDEF	5	28
			GGDEF_EAL	5	28
			HD-GYP	1	1

^a^ Genera numbers include any Sphingomonadales sp., Rhodobacteraceae sp., Rhizobiales sp., and Caulobacteraceae sp. designations as one each

### Identification of chromosomal origins of replication

The origin of replication (*ori*) was identified for each chromosome using Ori-Finder and default settings [41]. The ptt files were generated (https://github.com/sgivan/gb2ptt#gb2ptt) from gbff files, downloaded from NCBI on 23 April 2019. Only chromosomes with one unambiguously identified *ori* were subsequently included in the investigation, which reduced the dataset (Table 1). The terminus of replication (*ter*) was assumed to be opposite *ori* on the circular chromosomes [42].

### Phylogenetic analysis

RpoB sequences (PF05000, RNA polymerase Rpb1, domain 4) were downloaded for the members of each order and their NCBI identifiers were determined. Alignments were done using MAFFT with L-INS-i option [43] in Geneious version 11.0.5 [44]. Phylogenetic trees were reconstructed using IQ-TREE version 2.1.4 [45], with the best substitution matrix identified using ModelFinder. The robustness of the analysis was tested using a bootstrap test (1000 replicates) [46] and a hill-climbing nearest-neighbor interchange search [45, 47]. Trees were modified and annotated in iTOL version 5 [48].

## Results

### Occurrence of c-di-GMP-modulating domains

**Fig. 1:**
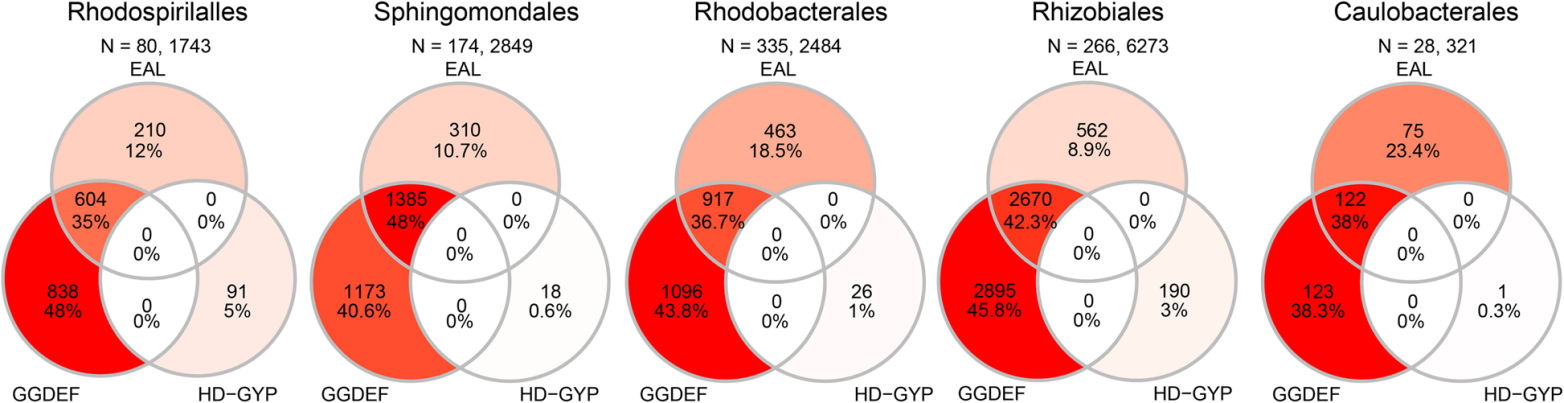
Numbers of sequences with GGDEF, EAL or HD-GYP sequences in the five orders. The number of genomes and the total number of sequences for each order are above the diagrams. The Venn diagrams show the numbers of sequences with both GGDEF and EAL domains in the corresponding overlapping circles. The coloration is a gradient from the highest (red) to lowest (white) values within each order

We quantified the genes encoding the domains associated with c-di-GMP synthesis and degradation in members of the five alphaproteobacterial orders. This included those that contained one of the GGDEF, EAL, or HD-GYP domains or both GGDEF and EAL domains. The GGDEF and GGDEF_EAL sequences accounted for the highest proportions in all five orders at 35–48%, followed by proteins containing an EAL domain that ranged between 8.9% and 23.4% of all sequences (Fig. 1). The HD-GYP domain-containing sequences made up the smallest share, accounting for only 0.3–5% of all sequences, and co-occurrence of GGDEF or EAL with an HD-GYP domain was not observed (Fig. 1). Each c-di-GMP-metabolizing domain was found almost exclusively once per sequence, but there were a few exceptions (Table S4).

**Fig. 2:**
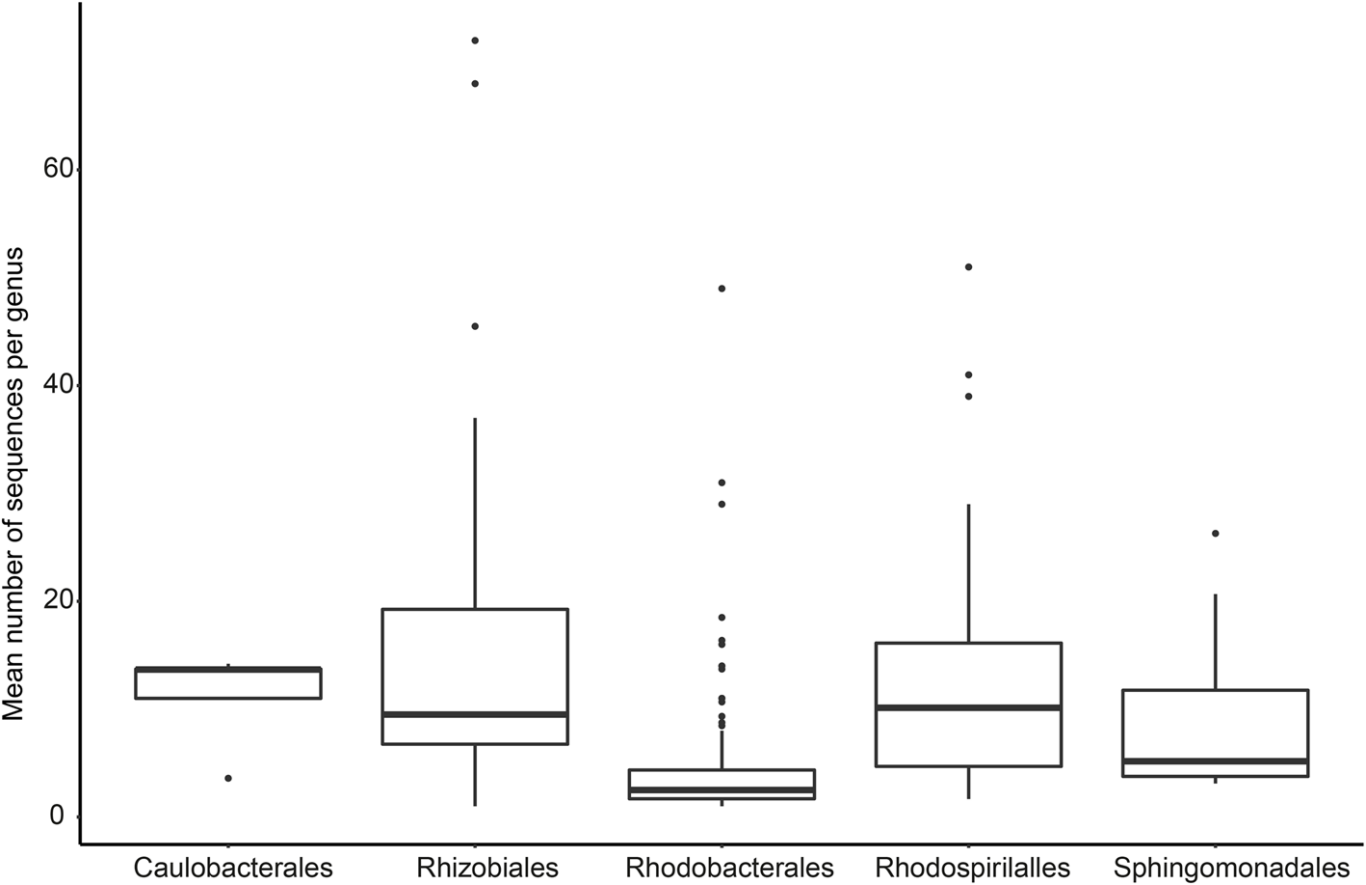
Mean number of c-di-GMP-metabolizing sequences per genome per genus in the different orders. The number of c-di-GMP-metabolizing genes in a genus was divided by the number of strains considered in the respective genus. The mean values from all genera of each order were used to make the box plot

Next, the numbers of c-di-GMP-metabolizing sequences in different genera were compared by calculating the mean number of sequences per genus (Fig. 2, Table S3). The c-di-GMP-metabolizing sequences per genus decreased from the Rhizobiales, Rhodospirillales, Caulobacterales, Sphingomonadales to the Rhodobacterales, but ranges of 1–72 (*Rhodomicrobium* and *Neorhizobium*), 1.7–51 (*Ferruginivarius* and *Thalassospira*), 3.6–14.2 (*Phenylobacterium* and *Caulobacter*), 3–25.4 (*Croceicoccus* and *Novosphingobium*), and 1–49 (*Salicibibacter* and *Roseibium*) were observed in the respective individual orders.

**Fig. 3:**
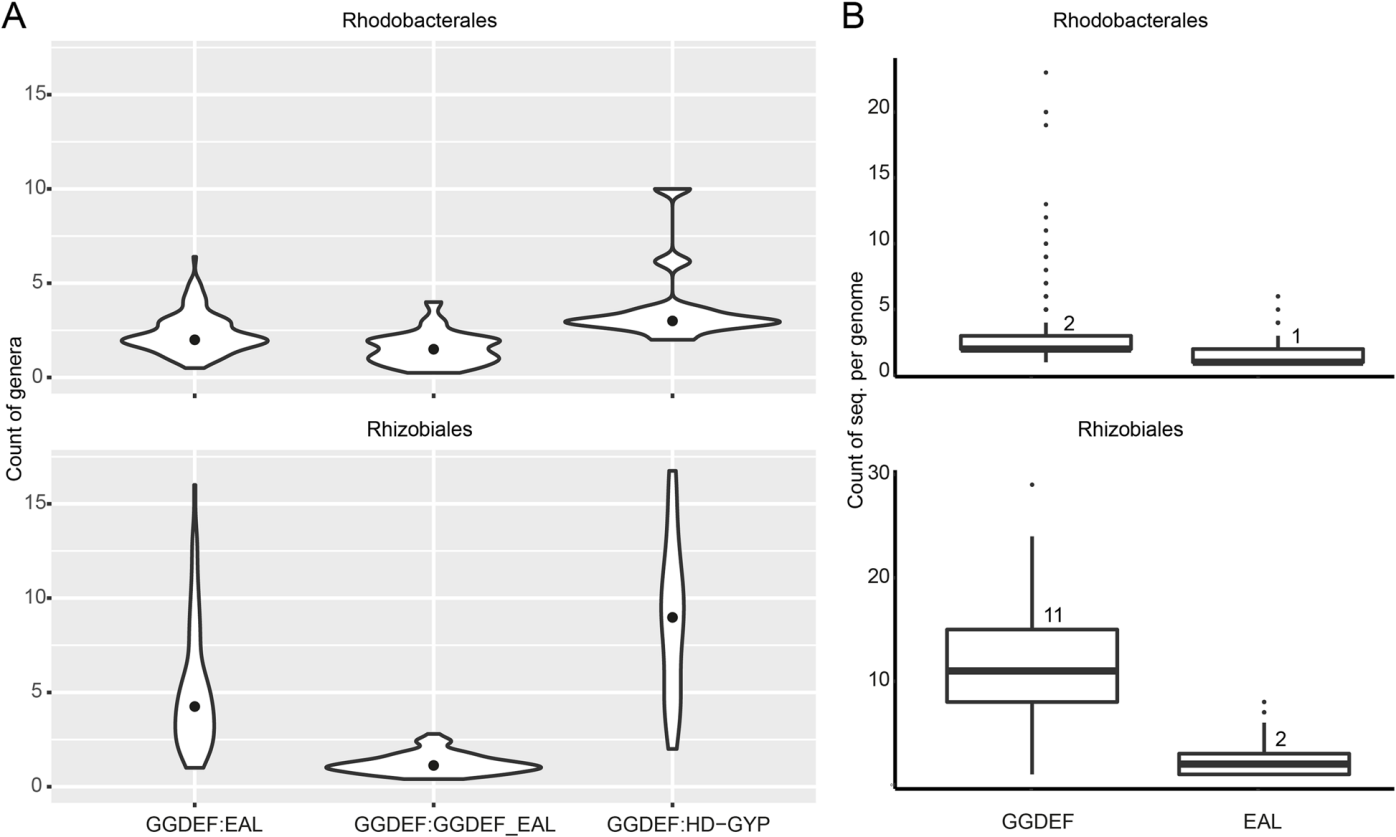
Numerical relationships among c-di-GMP-metabolizing sequences. A. Ratios for GGDEF:EAL, GGDEF:GGDEF_EAL, and GGDEF:HD_GYP sequences for the orders Rhodobacterales and Rhizobiales. The ratios were calculated per genome and the mean per genus was plotted. The median is indicated by the black dot. B. Counts of sequences with only a GGDEF domain or only an EAL domain per genome. The median value (50% quantile) is given on top of each box

For the subsequent investigation of the numerical relationships among the various domains, all orders were analyzed (Figure S1), but due to the larger number of available sequences and therefore more unambiguous results, we focused in particular on the Rhodobacterales and Rhizobiales. Examination of the per genus ratios of genes encoding synthesizing enzymes to those encoding hydrolyzing enzymes, i.e., GGDEF:EAL, revealed that this ratio was always 2 or higher (Fig. 3A). However, the ratio was more consistently close to 2 across the Rhodobacterales (0.5–6) as compared to the Rhizobiales (1–16), where there were more frequently higher numbers of GGDEF sequences and more variation among members of this order. When the numbers of GGDEF and EAL domain sequences per genome were examined (Fig. 3B), we found that the medians were 2 and 11 for GGDEF sequences and 1 and 2 for EAL sequences in the Rhodobacterales and Rhizobiales, respectively. This again shows that genes encoding the synthesizing enzymes occur more frequently than those encoding hydrolyzing enzymes in both orders. The GGDEF:GGDEF_EAL ratios peaked at 1 in the studied orders except the Rhodobacterales where more variability was observed and a higher proportion of members showed higher ratios (Fig. 3A, Figure S2). Interestingly, the relationships of the GGDEF:EAL and GGDEF:GGDEF_EAL ratios showed opposite patterns in the Rhodobacterales and Rhizobiales. While the GGDEF:EAL ratios were less variable and most consistently at 2 in the Rhodobacterales, there was much greater variability in the Rhizobiales. Conversely, there was more variability in the GGDEF:GGDEF_EAL ratios in the Rhodobacterales but a distinct peak at 1 in the Rhizobiales. The relationship of GGDEF:HD-GYP domains was found to be fairly consistent at 2.5:1 in the Rhodobacterales but highly variable in the Rhizobiales (Fig. 3A).

The large variability in numbers of c-di-GMP-metabolizing proteins among organisms stimulated us to investigate their evolutionary relationships. Therefore, the number of c-di-GMP enzymes present in different species was evaluated in a phylogenetic context (Figure S4). Some closely related groups were found in which the numbers of c-di-GMP genes were similar. In the Rhizobiales there was a large cluster in which the c-di-GMP-metabolizing gene numbers were elevated, and which consisted of several genera, including *Devosia, Fulvimarina* and *Rhizobium*. Smaller additional clusters with increased c-di-GMP numbers that were less closely related were also observed. In the Rhodobacterales, the closely related genera *Stapia* and *Labrenzia* stood out with their high c-di-GMP-metabolizing gene numbers. A connection between phylogeny and c-di-GMP-metabolizing gene number could also be observed in the Rhodospirillales. Here there were three clusters of organisms that had increased gene numbers and one notable group was made up of three genera including *Magnetospirillum, Magnetovibrio* and *Telmatosprillum*. A clear connection between phylogenetic relationships and numbers of c-di-GMP-metabolizing genes was not observed in the Sphingomonadales, and it is difficult to make any statement for the Caulobacterales because of the lower genome and gene numbers.

### Relationship between gene numbers, genome size, and location of c-di-GMP-metabolizing genes on secondary chromosomes

There was a statistically significant positive correlation between chromosome size and the number of c-di-GMP-metabolizing genes in all five orders (Figure S5). We only included the largest replicon in this analysis, although c-di-GMP-metabolizing genes were also found on secondary chromosomes and extrachromosomal replicons. In five genomes from different genera of the Rhodospirillales, six genomes from three genera in the Sphingomonadales, five genomes from five different genera of the Rhodobacterales, 23 genomes from 13 genera of the Rhizobiales, and one genome of the Caulobacterales c-di-GMP-metabolizing genes were found outside of the largest replicon (Table S5). In *Nitrospirillum amazonense* CBAmc (Rhodospirillales), *Rhizobium* sp. NXC24 (Rhizobiales) and *Asticcacaulis excentricus* CB 48 (Caulobacterales) more c-di-GMP genes were found on the second-largest replicon and in *Paracoccus denitrificans* PD1222 (Rhodobacterales) equal numbers of c-di-GMP-metabolizing genes were found on the largest and second-largest replicons.

**Fig. 4:**
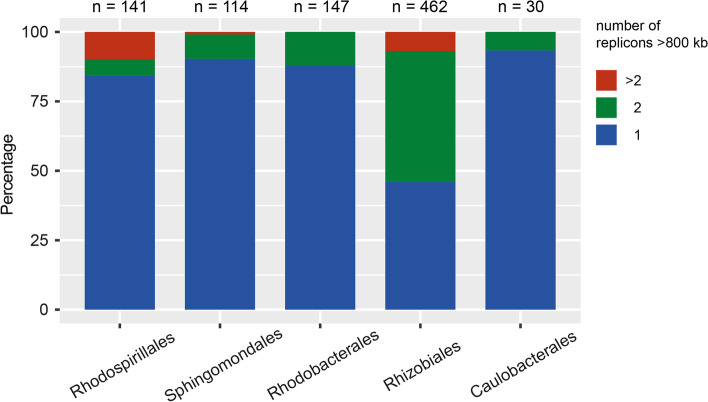
Proportions of genomes with one, two or more than two replicons > 800 kb in the five orders. The total numbers of genomes in each order are above the plot

Secondary chromosomes (defined as replicons > 800 kb that are not the largest replicons in the genome) contain genes that evolve faster [49] and are more common in the Rhizobiales (Fig. 4). We investigated if c-di-GMP-metabolizing genes were found outside of the largest chromosome more often when secondary chromosomes were present. We found that only a small fraction of the genomes examined in this study had secondary chromosomes in four of the orders (14.9% or 21 genomes of the Rhodospirillales, 8.8% or 10 genomes of the Sphingomonadales, 10% or 15 genomes of the Rhodobacterales, and 6.7% or 2 genomes of the Caulobacterales) whereas this was higher for the Rhizobiales (44% or 204 genomes). There were c-di-GMP-metabolizing genes on the secondary chromosomes in all orders and these accounted for 21.3, 21.1, 30, 31.7 and 73.3% of all c-di-GMP-metabolizing genes in the Rhodospirillales, Sphingomonadales, Rhodobacterales, Rhizobiales, and Caulobacterales, respectively. We note that the high percentage of c-di-GMP-metabolizing genes identified on secondary chromosomes in the Caulobacterales is based on only two genomes. Overall, the results indicate that the presence of secondary chromosomes did not result in a greater proportion of c-di-GMP-metabolizing genes located there.

### Chromosomal organization patterns of c-di-GMP-metabolizing genes

**Fig. 5:**
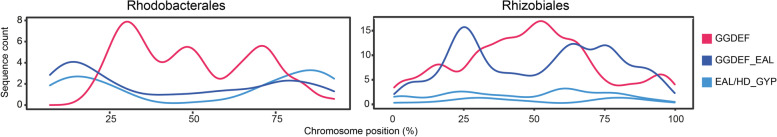
Chromosomal locations of c-di-GMP-metabolizing genes. Cumulative distributions of c-di-GMP-metabolizing genes on the chromosomes of Rhodobacterales and Rhizobiales, with lengths normalized to 100% where *ori* is at 0% and *ter* is at 50%. The color-coded lines represent the estimate of the kernel density. Only closed genomes with one unambiguously determined *ori* were used in this analysis

As discussed above, location on the chromosome can affect gene expression. We therefore wanted to examine the localization of c-di-GMP-metabolizing genes on chromosomes relative to the origin (*ori*) and terminus (*ter*) of replication. No obvious trend was observed in the Rhodospirillales, while GGDEF and GGDEF_EAL sequences seemed less prevalent near *ter* in the Sphingomonadales (Figure S6). The number of genes included in the analysis for the Sphingomonadales EAL group and all groups for the Caulobacterales were so low that patterns might not be obvious even if present. However, interesting patterns were evident in the Rhizobiales and Rhodobacterales (Fig. 5). In the Rhodobacterales the EAL and GGDEF_EAL sequences were predominately found near o*ri* whereas GGDEF sequences were predominately not close to *ori* and showed a tri-modal distribution with peaks mid-way between *ori* and *ter* and around *ter*. In the Rhizobiales, clear patterns were observed for the GGDEF and GGDEF_EAL sequences, which both showed multiple peaks but with opposing patterns. The distribution of the GGDEF sequences showed three peaks, with the largest near *ter* and two smaller peaks near *ori*. The GGDEF_EAL sequences peaked where the GGDEF sequences were lowest, mid-way between *ori* and *ter*. Although there were far fewer sequences, the Rhizobiales HD-GYP group showed a similar trend as the GGDEF_EAL sequences, while there was no obvious pattern for the EAL sequences.

Comparison of the similarities of distributions among the groups of genes indicated that the Rhodobacterales EAL and GGDEF_EAL genes were similarly distributed (two-sample Kolmogorov–Smirnov test; *p*-value = 0.16) while the EAL and GGDEF as well as the GGDEF and GGDEF_EAL pairs were distributed differently (two-sample Kolmogorov–Smirnov test; p-values = 0.009 and 0.0008, respectively). The Rhizobiales GGDEF and GGDEF_EAL genes were also distributed differently (two-sample Kolmogorov–Smirnov test; *p*-value = 0.04).

### Additional domains on c-di-GMP-metabolizing proteins

It has previously been documented that proteins with c-di-GMP-metabolizing domains frequently contain additional domains [20], hereafter referred to as auxiliary domains, which presumably function in many cases to regulate the c-di-GMP-related enzymatic activities. Only the Rhodobacterales and Rhizobiales are discussed in detail here because of the larger numbers of sequences available for these orders, but similar trends were also observed in the other three (Table S6, Figure S7). Auxiliary domains were associated with all four c-di-GMP sequence groups and there were 101 different auxiliary domains found across all five orders and sequence groups. We note that the auxiliary domains analyzed here are those that are identified and specified in databases but recognize that some of the sequences will have uncharacterized domains that are not captured there. We plotted the length of EAL-containing sequences and this showed that all those with identified auxiliary domains were > 375 amino acids long (Figure S8). The proportions of those without identified auxiliary domains that were < 375 amino acids long were 49% in the Rhizobiales and 82% in the Rhodobacterales, indicating that some of these proteins likely contain auxiliary domains but these remain to be recognized and annotated in the sequence databases. The same analysis with GGDEF sequences revealed that all sequences containing identified auxiliary domains were > 275 amino acids long (Figure S8). The proportions of those without identified auxiliary domains that were < 275 amino acids long were 13% in the Rhizobiales and 30% in the Rhodobacterales and, therefore, most of these sequences likely also contain currently unannotated auxiliary domains.

**Fig. 6:**
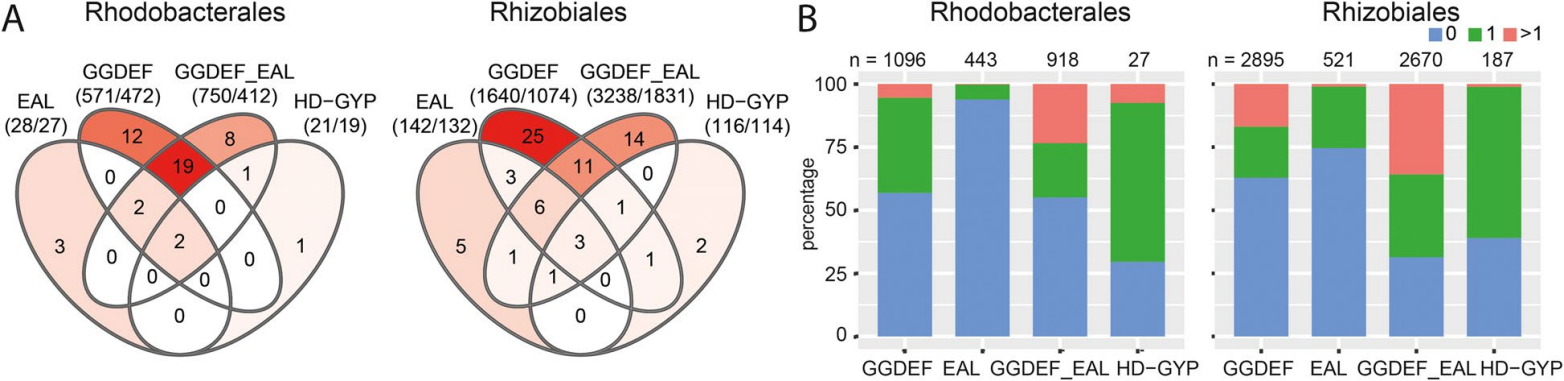
Occurrence of auxiliary domains on c-di-GMP-metabolizing proteins of the different enzyme groups. A. Numbers of different auxiliary domains that can be found for each group and shared among groups. The first number below the group identification (EAL, GGDEF, GGDEF_EAL, HD-GYP) indicates the number of auxiliary domains in the respective group and the second number indicates the number of sequences these domains are found in. The c-di-GMP-metabolizing domains themselves are not included in this analysis. The color code of the Venn diagram represents the domain counts from the highest (red) to zero (white). B. Percentage of sequences with none, one, or more than one auxiliary domain. The number of sequences included in this analysis is given above the group identification. Repeated occurrence of a domain in a sequence was counted as one

In both the Rhodobacterales and Rhizobiales the GGDEF group had the highest variability among auxiliary domains, followed by GGDEF_EAL, EAL and HD-GYP sequences (Fig. 6A). However, this could be driven by the higher number of sequences containing GGDEF domains compared to other domains (Fig. 1). The GGDEF and GGDEF_EAL groups had the greatest overlap of auxiliary domains whereas there were only a few unique domains present with the EAL and HD-GYP domain-containing sequences. Overall, there were uniform distributions of sequences that contain none, one, or more than one auxiliary domain (Fig. 6B, Figure S7). The HD-GYP group had the highest proportion of sequences with auxiliary domains, followed by the GGDEF_EAL, GGDEF and EAL groups (Fig. 6B). The GGDEF_EAL group had the biggest proportion of sequences that had more than one auxiliary domain on individual proteins.

Some auxiliary domains were more commonly found in certain groups and some of these co-occurrences were conserved across the five orders (Tables S6 and S7). A previous study reported that cGMP-specific phosphodiesterases, adenylyl cyclases and FhlA (GAF) and Per-Arnt-Sim (PAS) were the most common auxiliary domains associated with GGDEF domains in various bacterial species [17]. The GAF domain is a sensory domain involved in light sensing and it and the PAS domain have been found in phytochromes [17, 50]. In our GGDEF sequences, the response regulator receiver (REC) domain and PAS domain variants dominated. Cognate histidine kinases modulate REC domain-containing proteins through their phosphorylation status via their kinase and phosphatase activities, which are themselves regulated by various signals. The phosphorylation status of the REC domain then controls the activity of the associated output domain (e.g., GGDEF). In the EAL group REC domains, CSS-motif (Pfam PF12792) domains and GAF_2 domains were most common. CSS-motif domains are known for roles in redox sensing [17]. The Caulobacterales EAL sequences were an exception, because these were most often associated with histidine kinase and phosphotransferase domains that act upstream of REC domains in histidyl-aspartyl phosphorelay systems. In the GGDEF_EAL sequences, the PAS subfamilies PAS_3, PAS_4, PAS_7 and PAS_9, as well as the MHYT domain were most common. The MHYT domain consists of six transmembrane segments and it has been suggested to function in O_2_, NO and CO sensing [51]. In the HD-GYP sequences HD_5 and two domains of unknown function, DUF3369 and DUF3391, were the most prevalent.

**Fig. 7:**
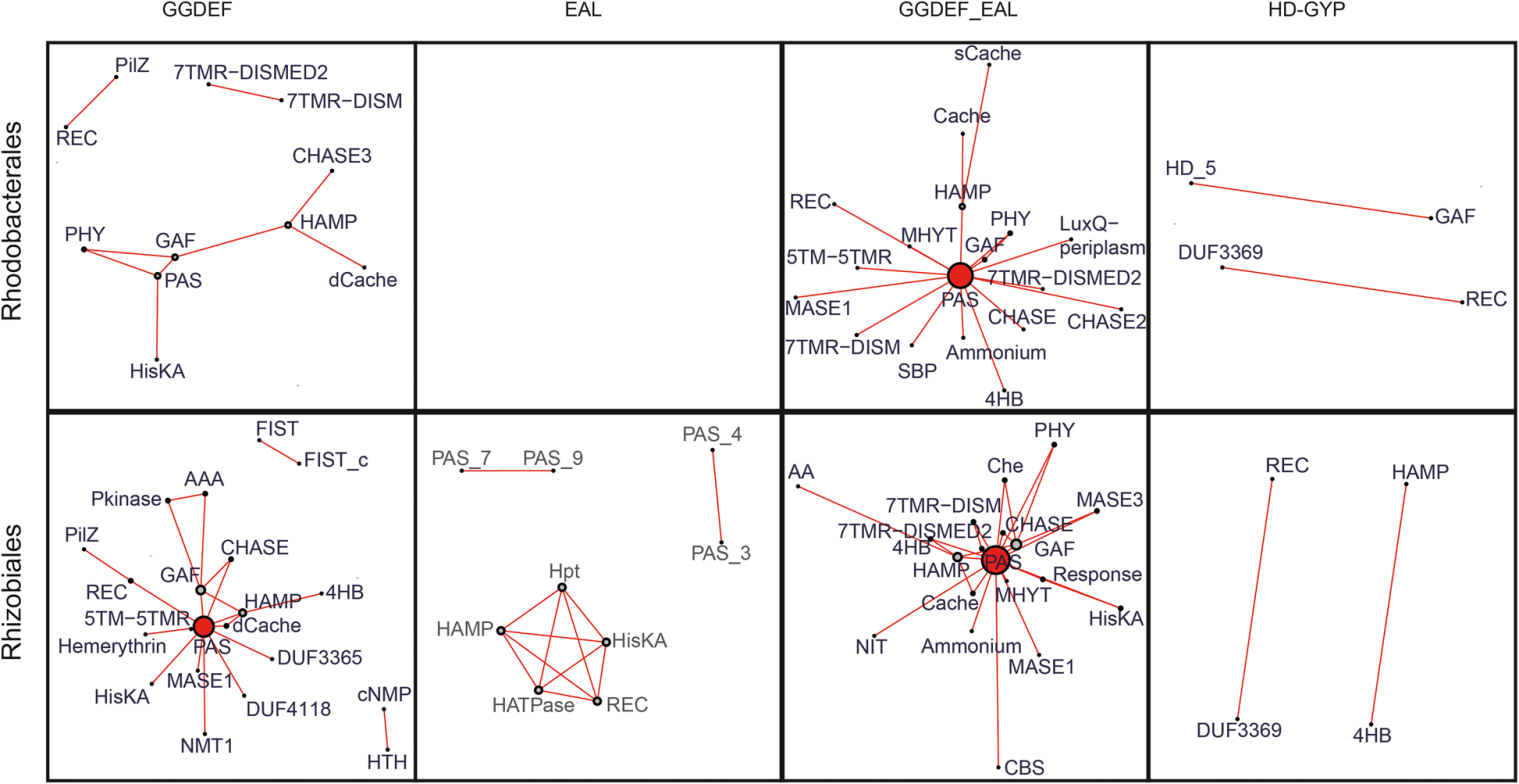
Weighted graphs representing the co-occurrences of auxiliary domains with c-di-GMP-metabolizing sequences. Auxiliary domains occurring together are connected by lines with the size and red color of the node indicating higher frequency of co-occurrence with other domains. Lengths of edges represent the number of times the connected domains co-occur, and the sizes of the points indicate the number of times these domains occur. All full domain names are provided in Table S8

Despite detailed knowledge on the structure and function of DGCs and PDEs, it has remained challenging to assign physiological roles to individual proteins. Analysis of the co-occurrence of additional domains might aid in assigning those roles. Therefore, we next investigated which additional domains occurred together and constructed co-occurrence networks (Fig. 6, Table S8). We focused on the Rhodobacterales and Rhizobiales because more sequences with more than one auxiliary domain were available for these orders. Most of the Rhodobacterales GGDEF sequences that had more than one auxiliary domain had co-occurrences of two specific auxiliary domains (Fig. 7). Exceptions were phytochrome (PHY), PAS, GAF, histidine kinase, adenylate cyclase, methyl-accepting protein and phosphatase (HAMP) domains, which co-occurred with two or three other domains. PAS domains were dominant in co-occurrences with many other domains in the GGDEF and GGDEF_EAL groups of both orders as well as the Rhizobiales’ EAL group (Fig. 7). Linkage of one domain with a variety of others creates complex patterns, such as found for the GGDEF sequences of both orders where calcium channels and chemotaxis receptors (dCache_1), GAF_2, HAMP and cyclase/histidine kinase-associated sensory extracellular (CHASE3) domains formed a network. The Cache and CHASE domains are extra-cytoplasmic sensory domains [52, 53] while the HAMP domain is usually found in integral membrane proteins that transmit conformational changes from periplasmic ligand-binding domains to cytoplasmic domains as part of histidyl-aspartyl phosphorelay signaling [54]. In the GGDEF_EAL sequences of both orders and the GGDEF sequences of the Rhizobiales, the PAS domains were notable because they are the domains connected with the most other auxiliary domains. Interestingly, the EAL sequences of the Rhizobiales had one cluster composed of the same domains that are most prevalent in the EAL sequences of the Caulobacterales (Table S7). These are the HisKA domain (activated via dimerization and able to transfer a phosphoryl group often as part of histidyl-aspartyl phosphorelay systems [55]), the Hpt domain that mediates phosphotransfer in histidyl-aspartyl phosphorelay systems [56], the HAMP domain, and HATPase that is found in multiple ATPases such as histidine kinases [57]. This shows that the EAL sequences, when linked to auxiliary domains, are often part of signaling cascades, especially in the Rhizobiales and Caulobacterales. The HD-GYP sequences showed two connections per order, one of which seemed to be conserved in the Rhodobacterales and Rhizobiales and consisted of the DUF3369 and REC domains.

## Discussion

### Associations with diverse auxiliary domains suggest a wide variety of signals affect DGC activity

Our analysis of the occurrence of the EAL, GGDEF, GGDEF_EAL and HD-GYP sequences in orders of the Alphaproteobacteria showed that the GGDEF and GGDEF_EAL domains made up the biggest proportions in all orders, followed by the EAL domains, while the HD-GYP domains accounted for the smallest share. Compared to results from a study on c-di-GMP-metabolizing gene distributions among prokaryotes, which found the overall proportions to be 50.4% GGDEF, 16.1% EAL and 33.5% GGDEF_EAL [11], the alphaproteobacterial orders have slightly lower GGDEF and higher GGDEF_EAL proportions. Moreover, the GGDEF and GGDEF_EAL sequences are associated with more different types of auxiliary domains and have a proportionally higher occurrence of auxiliary domains, respectively. This suggests that the GGDEF_EAL proteins more frequently respond to signals/stimuli, but the GGDEF-only proteins integrate a broader variety of signals. Thus, since GGDEF domain sequences are more abundant and seem to have more and more diverse auxiliary domains than PDE domain sequences, it could be that the synthesis of c-di-GMP is mainly controlled in response to extracellular and intracellular signals while its degradation is more unspecific. Since the GGDEF_EAL sequences of the Rhizobiales, like the EAL sequences of the Rhodobacterales, show a lower diversity of auxiliary domains, they too could be responsible for unspecific degradation while increases in c-di-GMP are more regulated. However, we note that this analysis is limited by its reliance on detecting recognized auxiliary domains while it is likely that some of these proteins contain currently unrecognized auxiliary domains.

### Importance of EAL-type PDE domains in Proteobacteria

Proteins with only EAL domains outnumbered those with HD-GYP domains at least two-fold in all orders. This agrees with a previous analysis of these domains in several phyla where the Proteobacteria, with the exception of the Deltaproteobacteria, and Oligoflexia were the only investigated phyla in which EAL domains outnumbered HD-GYP domains [58]. The driving forces behind the trends for relative abundances of these two different types of PDEs are not clear and likely require a larger phylogenetic analysis to untangle. More information on the specific roles of individual proteins is also required. The possible activities of proteins with both GGDEF and EAL domains, which are even more abundant than PDEs without GGDEF domains, further complicates the situation.

### Shared genomic features of the Rhizobiales GGDEF_EAL and Rhodobacterales EAL sequences

Interestingly, multiple commonalities exist between the GGDEF_EAL sequences of the Rhizobiales and the EAL sequences of the Rhodobacterales. Both gene groups are biased for localization away from *ter*, and their relative abundances compared to GGDEF sequences are reversed in the two orders. The GGDEF:EAL ratio is very consistent in the Rhodobacterales but there is no such consistency in the Rhizobiales. Conversely, while the GGDEF:GGDEF_EAL ratios were more varied in the Rhodobacterales, they were much more consistent in the Rhizobiales. This could indicate that the roles of the EAL sequences in the Rhodobacterales are swapped with GGDEF_EAL sequences in the Rhizobiales. However, the hybrid nature of GGDEF_EAL sequences makes this difficult to conclude. The two activities can be switched, e.g., by dimerization, which is required for GGDEF but not for EAL activity [15], or through regulation by auxiliary domains [14, 59, 60]. However, a study of the conservation of amino acid patterns showed that the catalytic activity in hybrid sequences is most often preserved in both domains or only in the EAL domain [16]. Future studies must show whether the Rhizobiales hybrid sequences have mainly retained EAL activity and thereby compensate for the lack of EAL sequences near *ori*, assuming they are involved in the same functions as the Rhodobacterales EAL sequences that are also positioned near *ori*. This could potentially be initially evaluated through a large-scale bioinformatic analysis of the enzymatic domains in the Rhizobiales GGDEF_EAL hybrids to look for conservation of known critical residues required for DGC and PDE activity.

### Conserved chromosomal positioning

In the two orders with the most available data, the Rhodobacterales and Rhizobiales, there is a clear conservation of the Rhodobacterales EAL- and GGDEF_EAL*-* and the Rhizobiales GGDEF_EAL-encoding genes away from *ter* while the GGDEF-encoding genes are predominant on the *ter*-proximate half of the chromosome in both orders. Overall, the concentrations of GGDEF genes peak when the EAL and GGDEF_EAL genes in the Rhodobacterales and the GGDEF_EAL genes in the Rhizobiales drop. This could indicate that there is more c-di-GMP degradation in the cell near the *ori* and more synthesis near the *ter* in the Rhodobacterales, which could also apply to the Rhizobiales should it turn out that the hybrid sequences primarily act as PDEs (discussed above).

There are multiple potential effects caused by the chromosomal locations of specific genes. The observed chromosomal localization patterns revealed in this study might affect cellular c-di-GMP concentrations during the cell cycle. Genes that are close to *ori* are replicated earlier than genes that are close to *ter*, which leads to a temporary copy number imbalance between genes at these two locations [32]. In *B. subtilis*, the opposite location of two genes encoding components of a phosphorelay with respect to *ori* and *ter* leads to temporal copy number imbalances, and this allows spore formation to only take place at the end of the cell cycle when the balance between the regulators is restored [33, 34]. In *Vibrio cholerae*, moving genes from *ori* to *ter* and thus reducing their copy number during the cell cycle has an impact on growth and infectivity [61, 62]. Such copy number imbalances can be pronounced in organisms that initiate multiple rounds of DNA replication within individual cells, such as *Escherichia coli* [63], although there is no evidence this occurs in members of the alphaproteobacteria. Regardless, it is possible that the biased localizations of genes encoding c-di-GMP-metabolizing enzymes we observed could have some effects on cellular c-di-GMP concentrations through temporary copy number imbalances, but future experimental work is required to evaluate this.

Another effect of localization could be manifested through DNA methylation, where the chromosomal DNA changes from fully methylated to hemi-methylated during replication. This change in methylation can affect gene transcription. For example, the p1 promoter of the *ctrA* gene in *C. crescentus* is only active in the hemi-methylated state [36]. Thus, *ctrA*, which is localized near *ori*, is transcribed more during DNA replication because it is hemi-methylated right at the beginning of the cycle. However, any broad role of methylation in regulating transcription of genes encoding c-di-GMP-metabolizing enzymes is currently unknown and future work is required to investigate this possibility.

## Conclusions

C-di-GMP-metabolizing enzymes are very diverse, and the specific roles and functions of only a few of these proteins are known. In this study new patterns and common properties for these proteins were identified in members of the Alphaproteobacteria. We systematically examined gene occurrence, localization on the genome, and the presence of auxiliary domains. In the Rhodobacterales and Rhizobiales, the EAL and GGDEF_EAL sequences, respectively, are primarily located away from *ter* while GGDEF sequences are biased towards *ter*. Additionally, the EAL and GGDEF_EAL domain-containing sequences show lower diversity and occurrence of auxiliary domains compared to the GGDEF sequences. There are several known examples in which chromosome localization of genes is important, and this can manifest in different ways such as through changes in copy number and methylation status during the cell cycle. The patterns we found support the suggestion that the chromosomal localization of c-di-GMP-metabolizing genes is important in these bacteria. Our findings also support the notion that the synthesis of c-di-GMP is more regulated and responsive to a variety of specific signals whereas its degradation might be less regulated and dependent on different stimuli.

## Supplementary Information

**Additional file 1:** **Figure S1.** Numerical relationships between the number of GGDEF and EAL (GGDEF:EAL) sequences by genera. The ratios were calculated per genome and the mean per genus was plotted. **Figure S2.** Numerical relationships between the number of GGDEF and GGDEF_EAL (GGDEF:GGDEF_EAL) sequences by genera. The ratios were calculated per genome and the mean per genus was plotted. **Figure S3.** Numerical relationships between the number of GGDEF and HD-GYP (GGDEF:HDGYP) sequences by genera. The ratios were calculated per genome and the mean per genus was plotted. **Figure S4.** Numbers of c-di-GMP sequences in a phylogenetic context. Phylogenetic relationships are based on RpoB sequences. All alignments were done using MAFFT with LINS-i option. Bootstrap values based on 1000 replicates and hill-climbing nearest-neighbor interchange search were used. A. Rhizobiales. B. Caulobacterales. C. Rhodobacterales. D. Rhodospirillales. E. Sphingomonadales. **Figure S5.** Relationships between chromosome size and the number of encoded c-di-GMP enzymatic domains. Spearman's rank correlation was used to evaluate the significance. Only the biggest replicon, considered the main chromosome, was included in this analysis. **Figure S6.** Chromosomal locations of c-di-GMP-associated genes. Cumulative distributions of cdi-GMP-associated genes on the chromosomes, with lengths normalized to 100% where ori is at 0% and 100% and ter is at 50%. The red line indicates the estimate of the kernel density. In this analysis only closed genomes with one unambiguously identified ori were used. **Figure S7.** Secondary domains that are present along with the different c-di-GMP-associated enzyme groups. A. Shared and individual secondary domains. The c-di-GMP-modulating domains are not included in this analysis. The color code of the Venn diagram represents domain counts from highest(red) to zero (white). B. Number of sequences that have zero, one, or more than one secondary domain. **Figure S8.** Relationships between protein length and presence of detected auxiliary domains. The sequences with EAL and GGDEF domains were segregated based on the occurrence of auxiliary domains. The minimal amino acid lengths for proteins containing auxiliary domains (left panel) were identified as 375 for EAL proteins and 275 for GGDEF proteins (blue dashed lines). This threshold was then used to calculate the percentage of sequences without identified auxiliary domains that were shorter and longer than these minimal lengths (right panel).

**Additional file 2:** **Table S1.** R packages used for analyses.

**Additional file 3:** **Table S2.** Compilation of Pfam, EBI and NCBI sequence identifiers.

**Additional file 4:** **Table S3.** Sequence counts by genera. The mean number of sequences is calculated per genus. Blue indicates a "genus" that is not included in the analysis because it does not represent an actual genus.

**Additional file 5:** **Table S4.** Domains found in all c-di-GMP-associated protein sequences. The cyclic di-GMP-associated domains are indicated in light blue. Values >0 for other domains are indicated in red.

**Additional file 6:** **Table S5.** Count of c-di-GMP-associated genes on chromosomes and plasmids. The primary chromosome is defined as the largest replicon of a genome. A "c" at the start of a column name indicates information regarding the chromosome while "p" indicates plasmids. Blue coloration indicates those genomes that have c di GMP-associated genes on secondary chromosomes or extrachromosomal replicons.

**Additional file 7:** **Table S6.** Occurrence of secondary domains with cyclic di-GMP-modulating domain sequences.

**Additional file 8:** **Table S7.** Frequency of association of all secondary domains that co-occur with c-di-GMP-associated domains.

**Additional file 9:** **Table S8.** All domains found associated with one of the c-di-GMP-associated domains examined in this study with associated Pfam HTML paths and Pfam IDs.

## Data Availability

Data were withdrawn from the databases Pfam, EBI and NCBI. For identification hyperlinks or protein identifiers are supplied in the Supplementary Table S2 and in the Methods section.
