# Supplementary material for: Patterns of abundance, chromosomal localization, and domain organization among c-di-GMP-metabolizing genes revealed by comparative genomics of five alphaproteobacterial orders
Source: BMC Genomics. 2022 Dec 16;23:834. doi: 10.1186/s12864-022-09072-9 (PMC9756655; doi:10.1186/s12864-022-09072-9)
Supplement: Supplementary file 1 — Additional file 1: Figure S1. Numerical relationships between the number of GGDEF and EAL (GGDEF:EAL) sequences by genera. The ratios were calculated per genome and the mean per genus was plotted. Figure S2. Numerical relationships between the number of GGDEF and GGDEF_EAL (GGDEF:GGDEF_EAL) sequences by genera. The ratios were calculated per genome and the mean per genus was plotted. Figure S3. Numerical relationships between the number of GGDEF and HD-GYP (GGDEF:HDGYP) sequences by genera. The ratios were calculated per genome and the mean per genus was plotted. Figure S4. Numbers of c-di-GMP sequences in a phylogenetic context. Phylogenetic relationships are based on RpoB sequences. All alignments were done using MAFFT with LINS-i option. Bootstrap values based on 1000 replicates and hill-climbing nearest-neighbor interchange search were used. A. Rhizobiales. B. Caulobacterales. C. Rhodobacterales. D. Rhodospirillales. E. Sphingomonadales. Figure S5. Relationships between chromosome size and the number of encoded c-di-GMP enzymatic domains. Spearman's rank correlation was used to evaluate the significance. Only the biggest replicon, considered the main chromosome, was included in this analysis. Figure S6. Chromosomal locations of c-di-GMP-associated genes. Cumulative distributions of cdi-GMP-associated genes on the chromosomes, with lengths normalized to 100% where ori is at 0% and 100% and ter is at 50%. The red line indicates the estimate of the kernel density. In this analysis only closed genomes with one unambiguously identified ori were used. Figure S7. Secondary domains that are present along with the different c-di-GMP-associated enzyme groups. A. Shared and individual secondary domains. The c-di-GMP-modulating domains are not included in this analysis. The color code of the Venn diagram represents domain counts from highest(red) to zero (white). B. Number of sequences that have zero, one, or more than one secondary domain. Figure S8. Relationships between [file 12864_2022_9072_MOESM1_ESM.pdf]

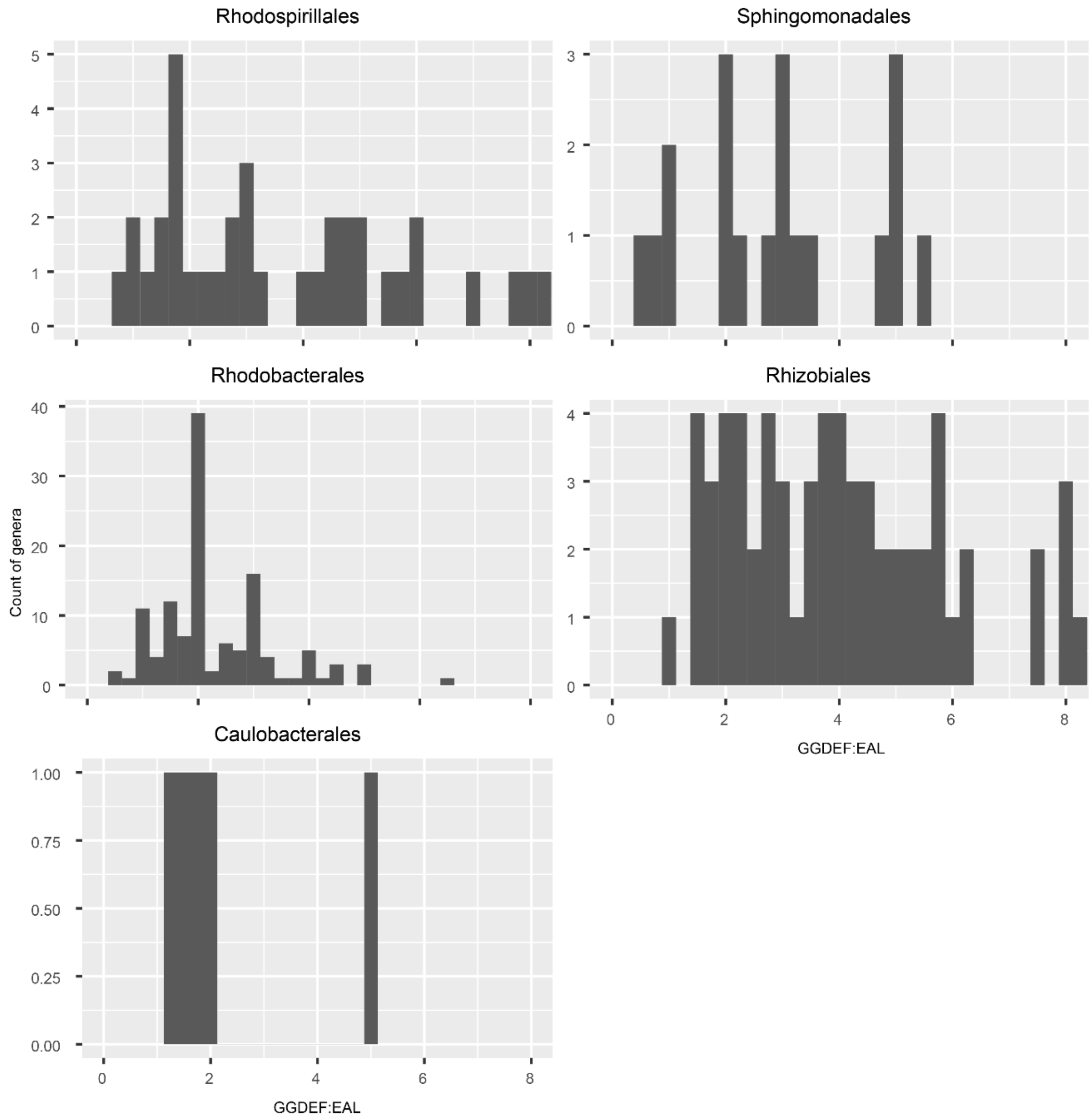

Figure S1. Numerical relationships between the number of GGDEF and EAL (GGDEF:EAL) sequences by genera. The ratios were calculated per genome and the mean per genus was plotted.

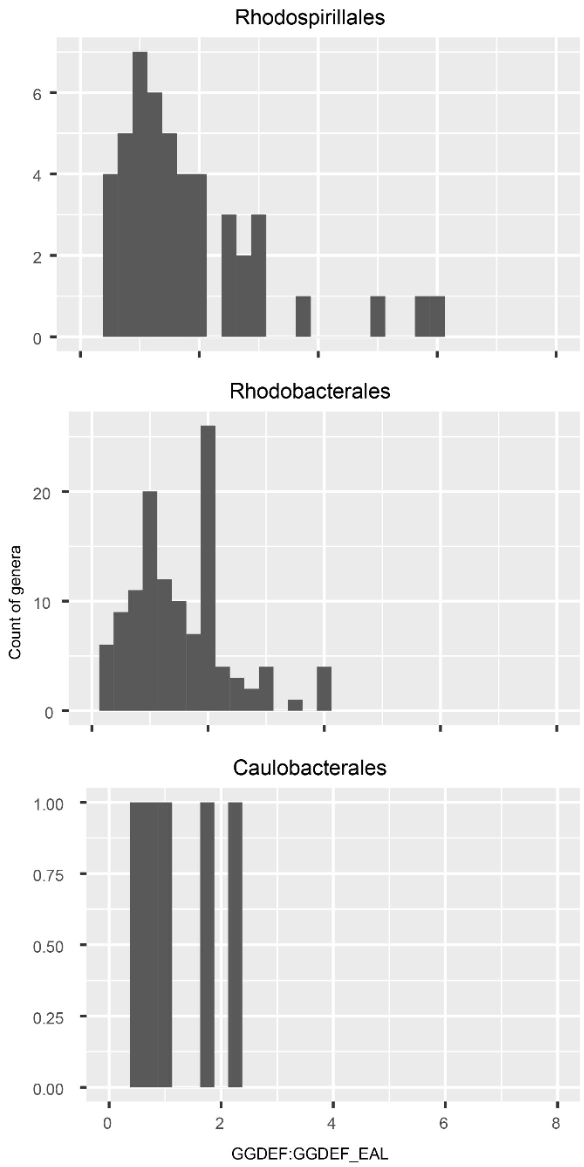

Figure S2. Numerical relationships between the number of GGDEF and GGDEF_EAL (GGDEF:GGDEF_EAL) sequences by genera. The ratios were calculated per genome and the mean per genus was plotted.

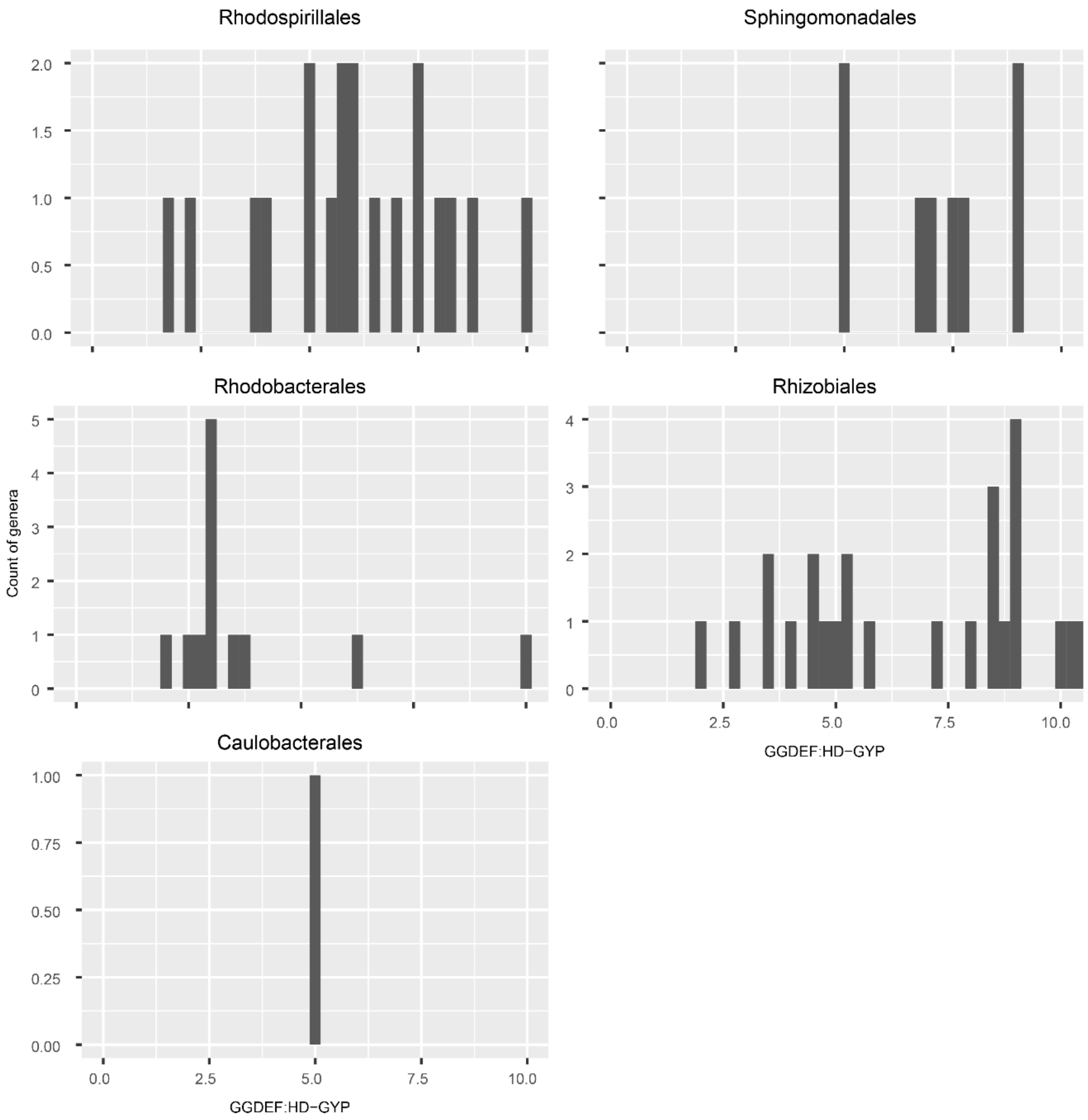

Figure S3. Numerical relationships between the number of GGDEF and HD-GYP (GGDEF:HD-GYP) sequences by genera. The ratios were calculated per genome and the mean per genus was plotted.

A

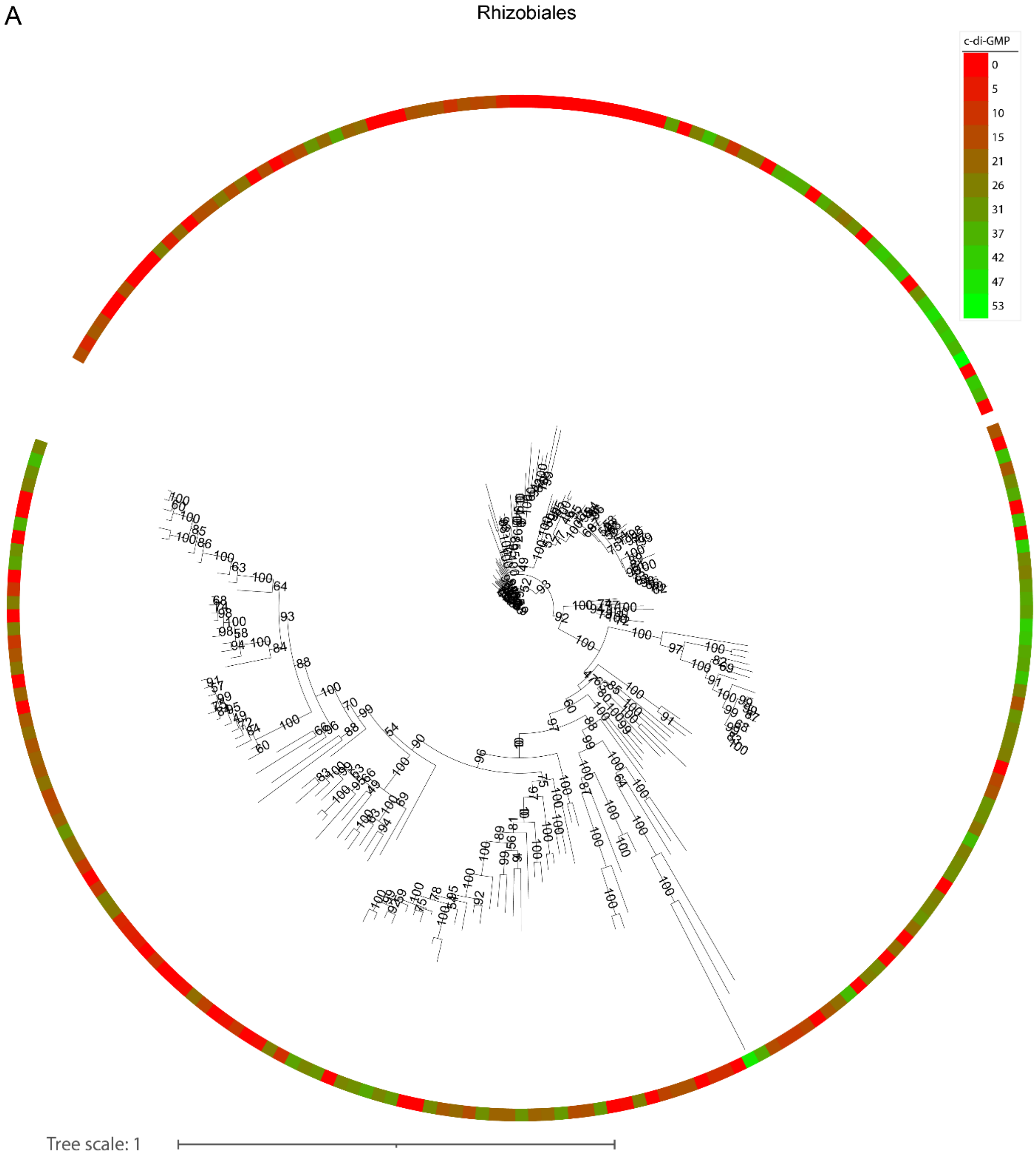

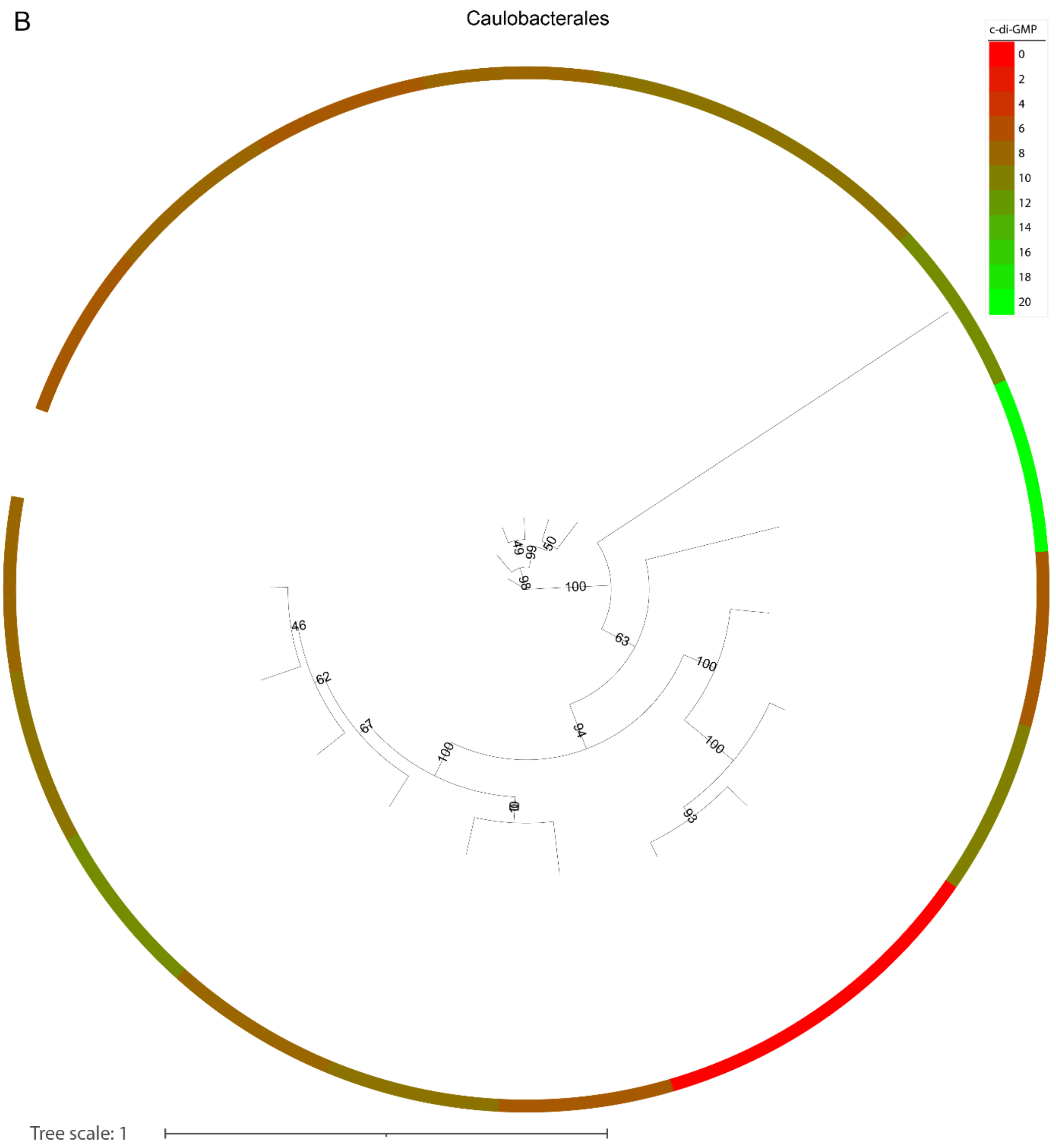
B
Caulobacterales
c-di-GMP
0
2
4
6
8
10
12
14
16
18
20
50
66
49
98
100
63
46
62
67
100
94
100
100
93
Tree scale: 1

C

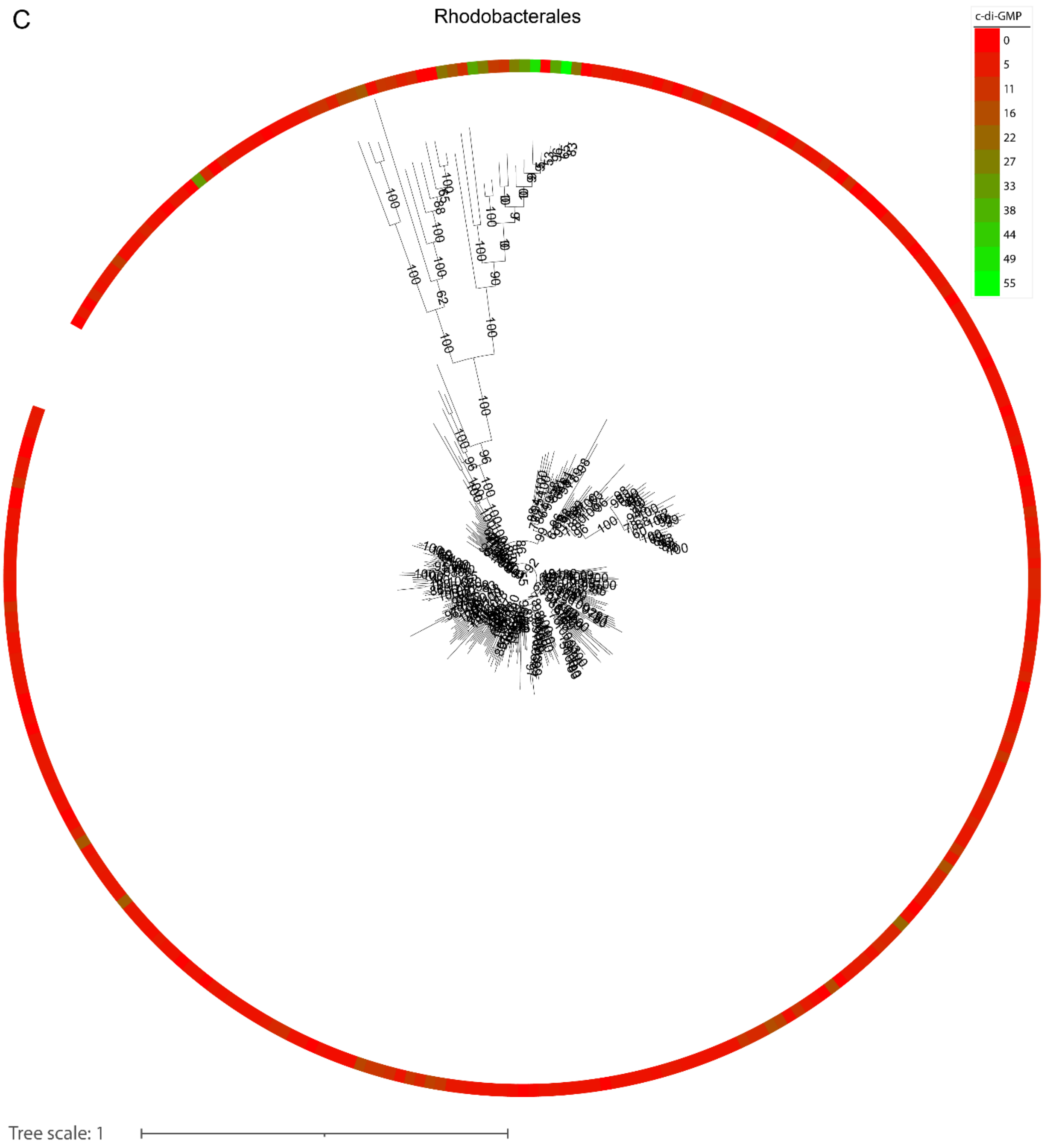

Rhodobacterales
c-di-GMP
0
5
11
16
22
27
33
38
44
49
55
Tree scale: 1

D

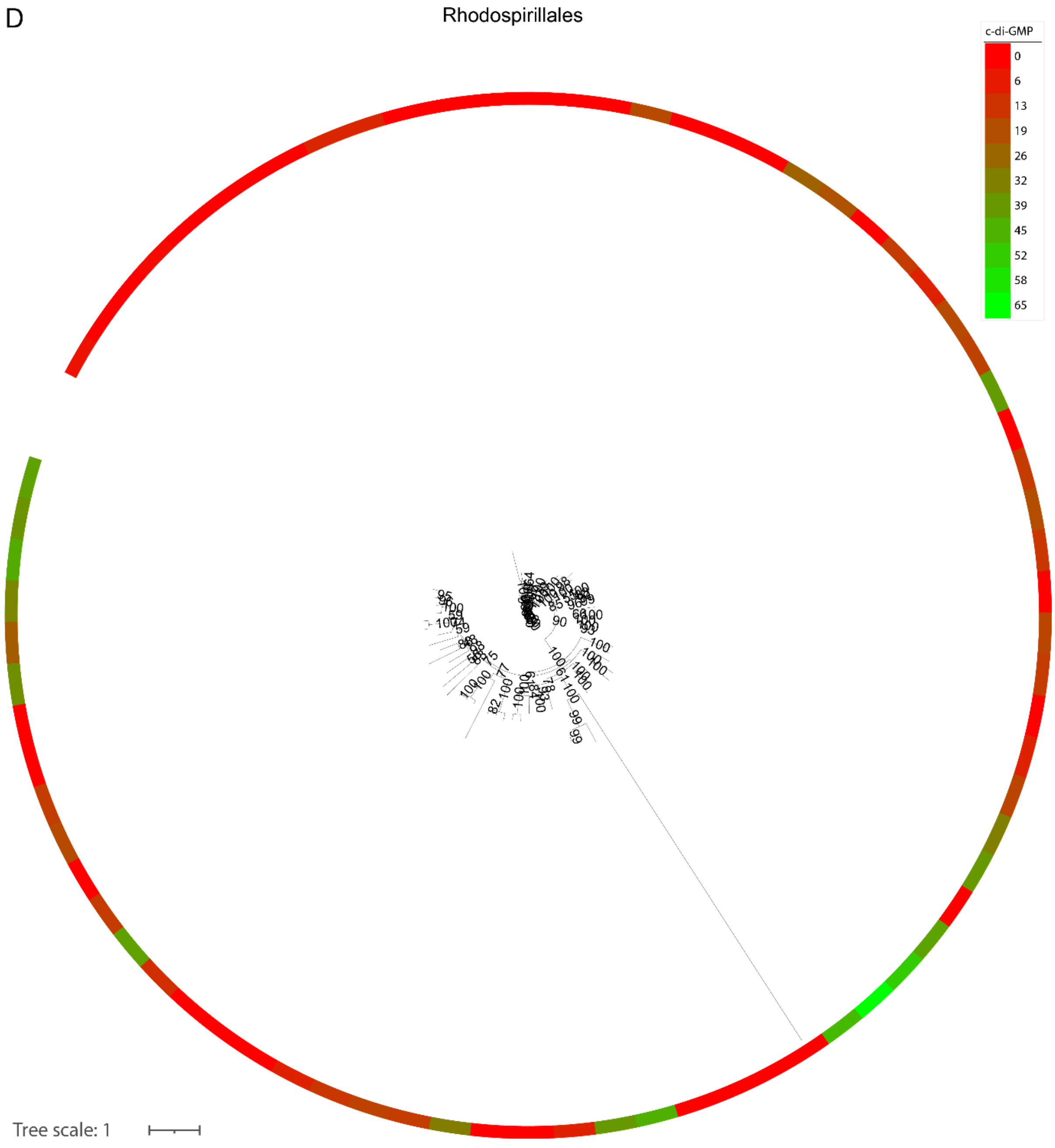
Rhodospirillales
c-di-GMP
0
6
13
19
26
32
39
45
52
58
65
Tree scale: 1

E

Sphingomondales

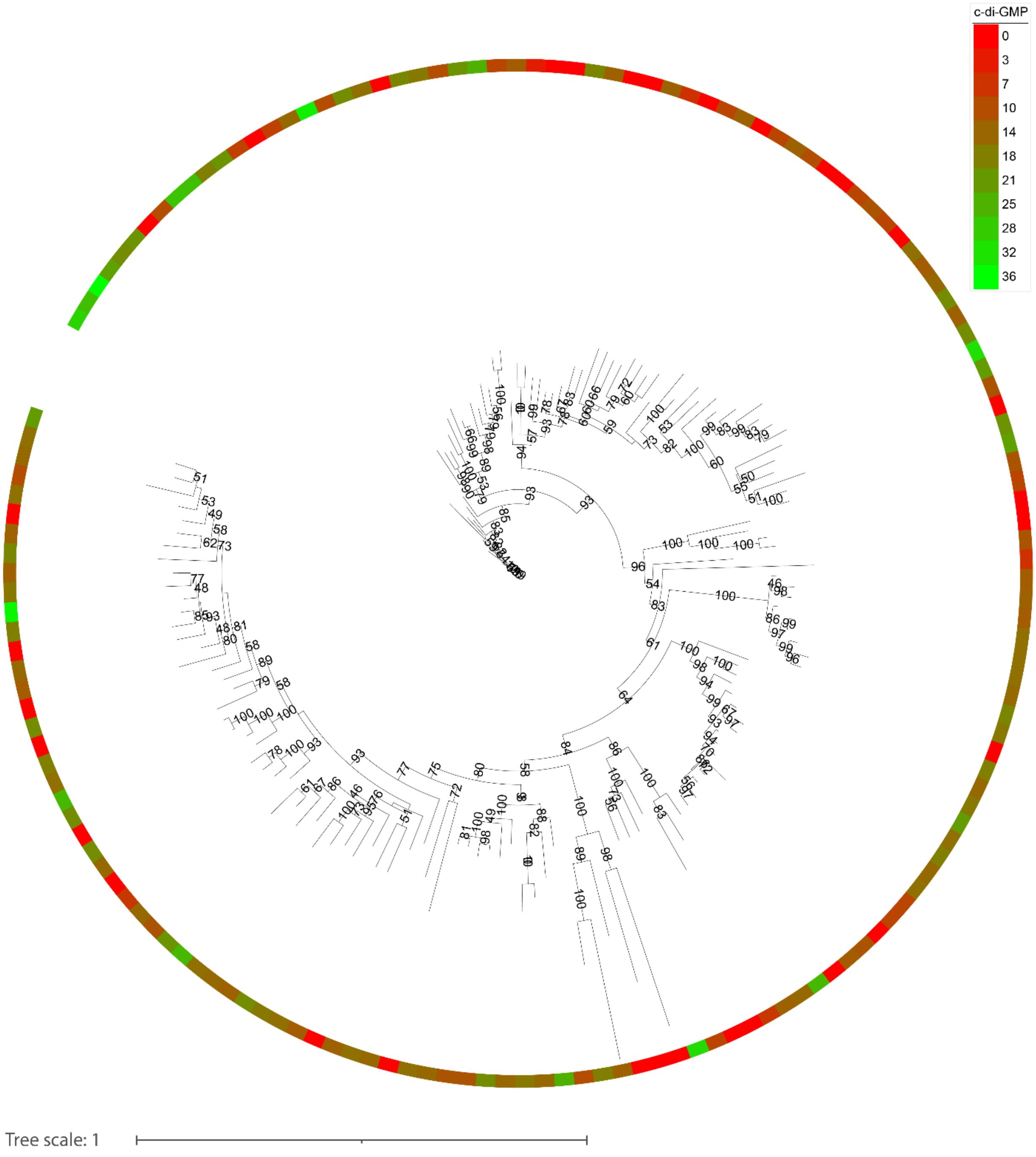

Figure S4. Numbers of c-di-GMP sequences in a phylogenetic context. Phylogenetic relationships are based on RpoB sequences. All alignments were done using MAFFT with L-INS-i option. Bootstrap values based on 1000 replicates and hill-climbing nearest-neighbor interchange search were used. A. Rhizobiales. B. Caulobacterales. C. Rhodobacterales. D. Rhodospirillales. E. Sphingomonadales.

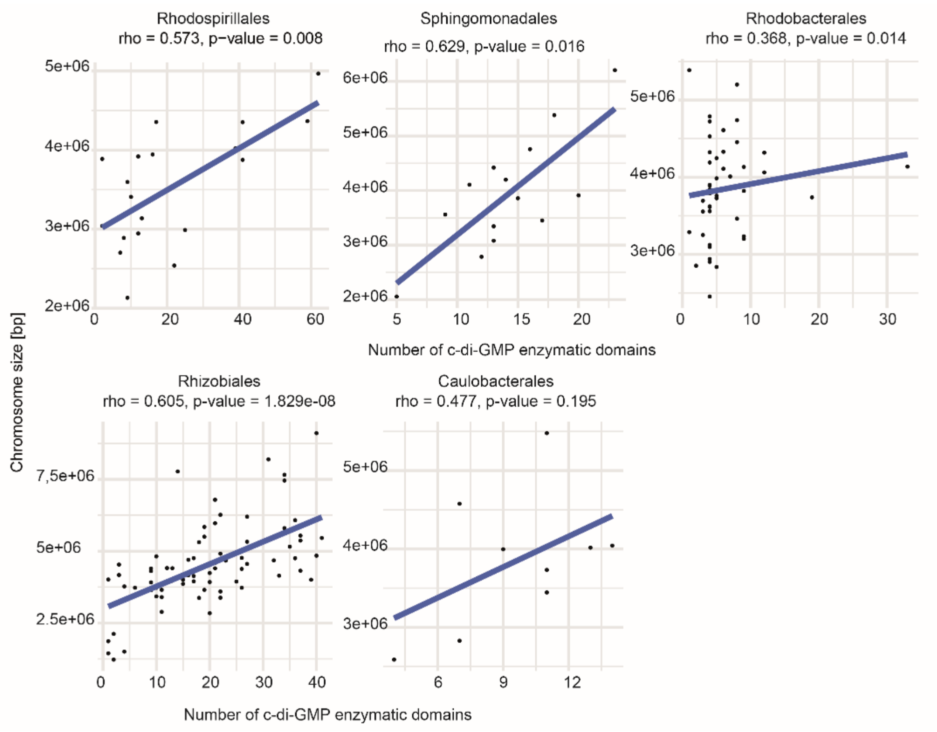

Figure S5. Relationships between chromosome size and the number of encoded c-di-GMP enzymatic domains. Spearman's rank correlation was used to evaluate the significance. Only the biggest replicon, considered the main chromosome, was included in this analysis.

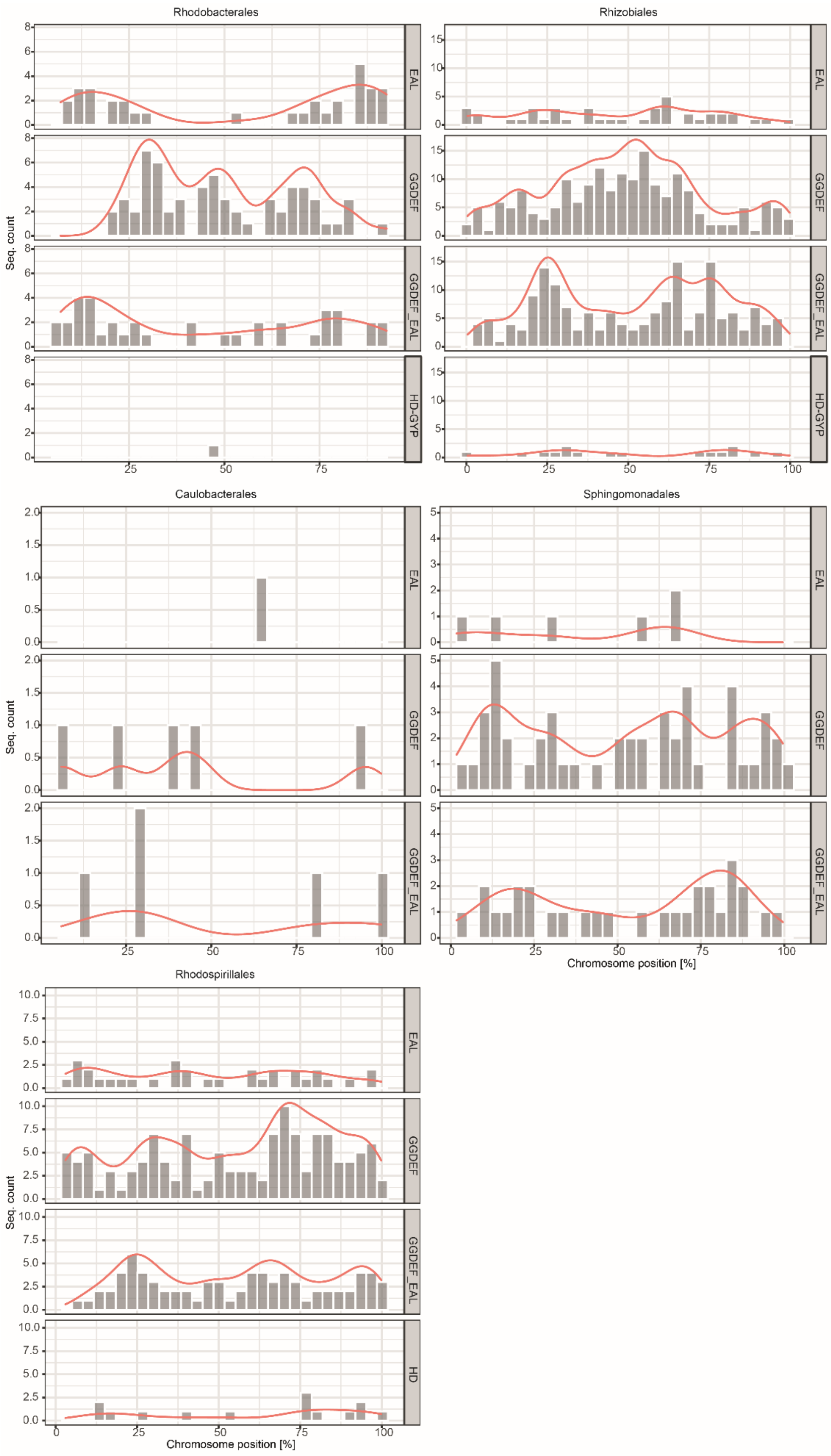
Rhodobacterales
Rhizobiales
Caulobacterales
Sphingomonadales
Rhodospirillales
EAL
GGDEF
GGDEF_EAL
HD-GYP
HD
Seq. count
Chromosome position [%]

Figure S6. Chromosomal locations of c-di-GMP-associated genes. Cumulative distributions of c-di-GMP-associated genes on the chromosomes, with lengths normalized to 100% where *ori* is at 0% and 100% and *ter* is at 50%. The red line indicates the estimate of the kernel density. In this analysis only closed genomes with one unambiguously identified *ori* were used.

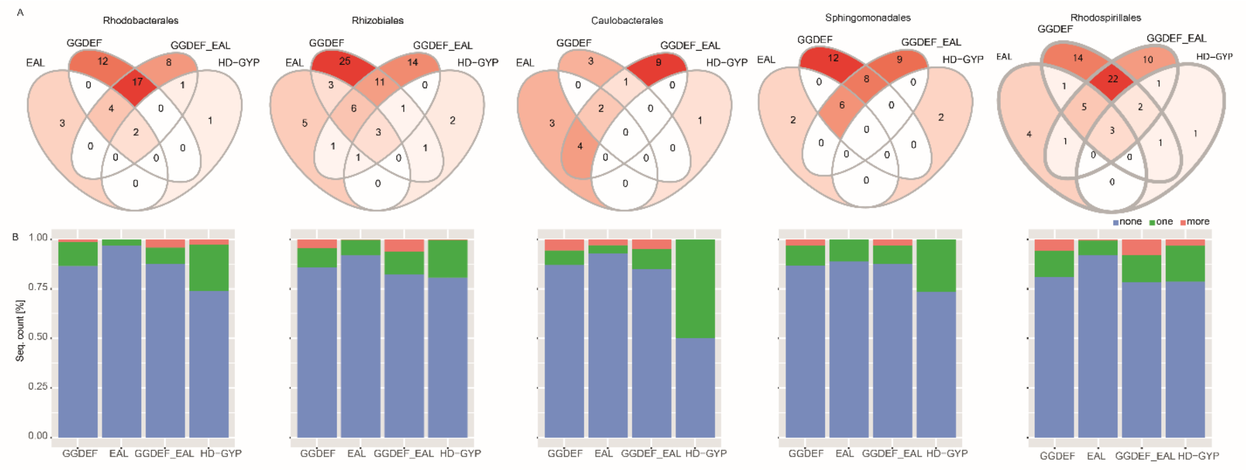

Figure S7. Secondary domains that are present along with the different c-di-GMP-associated enzyme groups. A. Shared and individual secondary domains. The c-di-GMP-modulating domains are not included in this analysis. The color code of the Venn diagram represents domain counts from highest (red) to zero (white). B. Number of sequences that have zero, one, or more than one secondary domain.

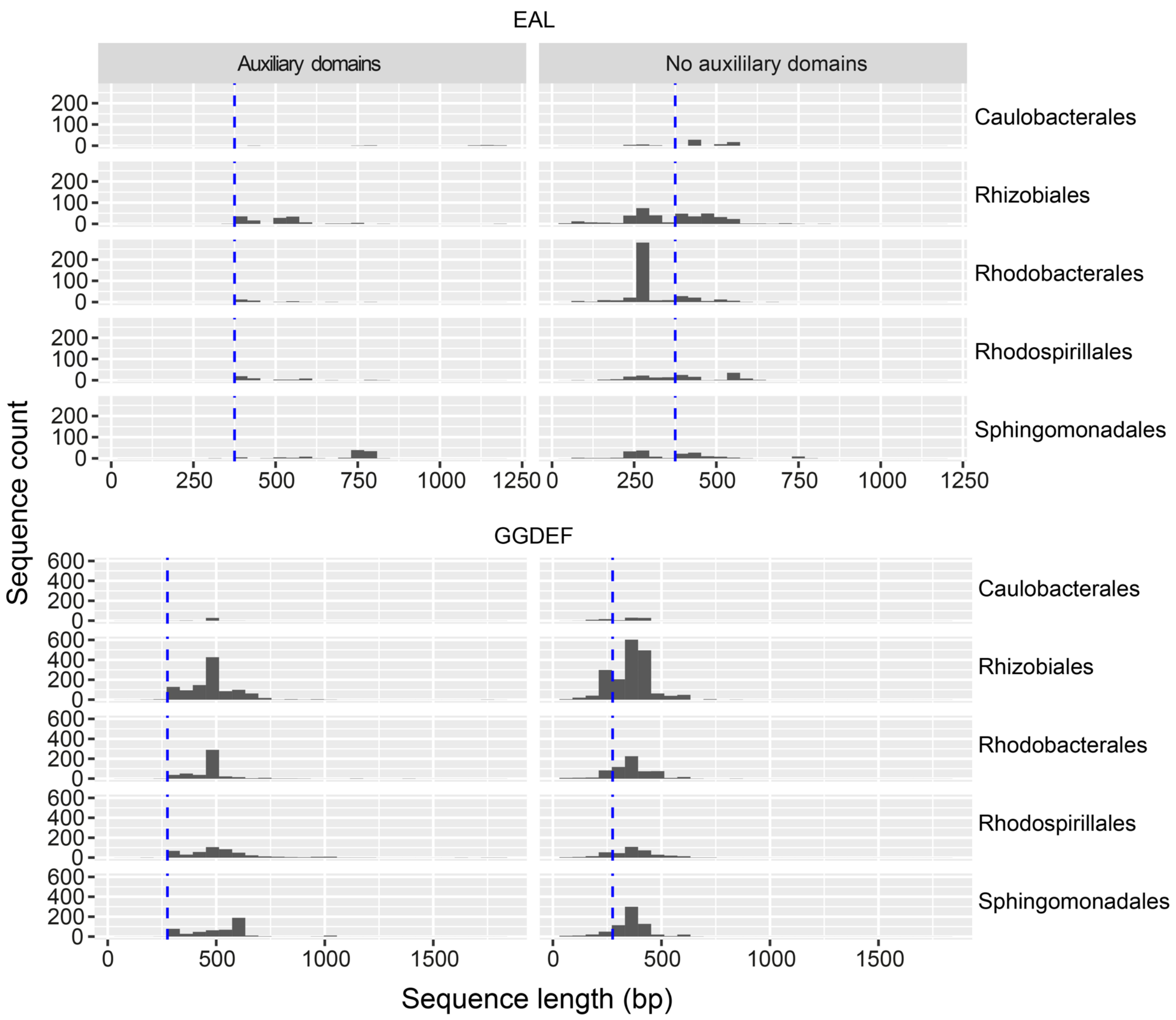

Figure S8. Relationships between protein length and presence of detected auxiliary domains. The sequences with EAL and GGDEF domains were segregated based on the occurrence of auxiliary domains. The minimal amino acid lengths for proteins containing auxiliary domains (left panel) were identified as 375 for EAL proteins and 275 for GGDEF proteins (blue dashed lines). This threshold was then used to calculate the percentage of sequences without identified auxiliary domains that were shorter and longer than these minimal lengths (right panel).
